# Dual Electrorheological and Magnetorheological Behaviors of Poly(N-methyl aniline) Coated ZnFe_2_O_4_ Composite Particles

**DOI:** 10.3390/ma15072677

**Published:** 2022-04-05

**Authors:** Hyun Min Kim, Ji Yoon Jeong, Su Hyung Kang, Hyoung-Joon Jin, Hyoung Jin Choi

**Affiliations:** Department of Polymer Science and Engineering, Inha University, Incheon 22212, Korea; khm9508@naver.com (H.M.K.); 22202250@inha.edu (J.Y.J.); suuhyung@naver.com (S.H.K.)

**Keywords:** electrorheology, magnetorheology, core-shell, zinc ferrite, poly(N-methyl aniline)

## Abstract

Magnetic/conducting polymeric hybrid core-shell typed zinc ferrite (ZnFe_2_O_4_)/poly(N-methyl aniline) (PMA) particles were fabricated and adopted as electrorheological (ER) and magnetorheological (MR) fluids, and their rheological properties were examined. Solvo-thermally synthesized ZnFe_2_O_4_ was coated with a conducting PMA through chemical oxidation polymerization. The size, shape, and chemical composition of the final core-shell shaped particles were scrutinized by scanning electron microscopy, transmission electron microscopy, and Fourier transform-infrared spectroscopy. The crystal faces of the particles before and after coating with PMA were analyzed by X-ray diffraction. The ZnFe_2_O_4_/PMA products were suspended in silicone oil to investigate the rheological response to electro- or magnetic stimuli using a rotating rheometer. The shear stresses were analyzed using the CCJ equation. The dynamic yield stress curve was suitable for the conductivity mechanism with a slope of 1.5. When magnetic fields of various intensities were applied, the flow curve was analyzed using the Hershel–Bulkley equation, and the yield stresses had a slope of 1.5. Optical microscopy further showed that the particles dispersed in insulating medium form chain structures under electric and magnetic fields. Via this core-shell fabrication process, not only spherical conducting particles were obtained but also their dual ER and MR responses were demonstrated for their wide potential applications.

## 1. Introduction

External field responsive smart fluids can be applied in many technological applications [1], such as intelligent hydraulic systems and smart robots because their rheological and physical behaviors can be changed by external electric and magnetic stimuli [2,3,4]. Under either an electric field strength (*E*) or magnetic field strength (*H*), the state of the electrorheological (ER) and magnetorheological (MR) fluid rapidly varies reversibly from liquid-like to solid-like within milliseconds due to structural chain development by the external fields [5]. When the external stimulus is removed, the smart fluid immediately reverts to its original liquid-like state with Newtonian behavior. The characteristics of an ER and MR fluid can differ according to the shape, size, electrical properties, magnetic properties, and dispersion medium of the dispersed particles [6]. Smart fluids have been used in various industrial applications based on their reversible and controllable properties [7]. ER fluids are suitable for applications that increase engine efficiency because of their short response time and simple mechanical engineering advantages. For example, devices such as robotics, haptic devices, automobiles, hydraulics, and fluid sealing, have been reported [8,9]. The industrial applications of MR fluids have been employed extensively in mechanical engineering devices, such as smart structures, mounts, dampers, hyperthermia, and clutches [10,11,12].

Insulating liquids, such as silicone oil and vegetable oil used as a medium for E/MR fluids, have low viscosity, high electrical resistance, and are non-magnetic. They are responsible for transporting solid particles in E/MR suspension systems [13]. For ER particles, they need to be semiconducting and polarizable, such as conducting polymers, inorganic oxides, and biopolymers [14,15,16]. ER fluids have a fast response and colloidal stability but insufficient yield stress under an electric field, limiting their applications in industry [17]. MR particles are magnetic particles with soft magnetic properties and almost no magnetic hysteresis. Hence, they can be controlled reversibly under a magnetic field [18,19,20]. Research on MR fluids has been continuous due to limitations, such as corrosion and dispersion stability and low reversibility of dispersed magnetic materials [21,22]. Carbonyl iron (CI) is a representative soft magnetic particle that has drawn attention because of its good saturation magnetization, but its high density causes sedimentation problems.

The MR effect and dispersion stability of various magnetic particles have been reported. In particular, studies on the improved sedimentation stability of ferrite particles with low density as a countermeasure to replace CI particles have been reported [23,24]. Metal ferrites, such as nickel ferrite, zinc ferrite (ZnFe_2_O_4_), and cobalt ferrite, have potential MR applications [25,26]. Among the methods of synthesizing zinc ferrite, the solvothermal process produces products with high saturation magnetization and crystal stability [27]. When conducting polymers are chosen as the shell in zinc ferrite, a dual response to an electric and magnetic field can be expected [28,29]. The semiconductor magnetite/polymer composites can control the particle size and density in a system with a core/shell structure, and functional materials can be produced through single or multiple coatings [30]. Among the conducting polymers that impart an ER effect, aniline-based polymers have characteristics such as excellent electrochemical performance, environmental stability, and price competitiveness. Hence, studies on polyaniline and its derivatives are being conducted [31]. In this case, the coating thickness of the conductive polymer can be controlled by adjusting the amount of the monomer [32].

In this study, core–shell-type particles showing ER and MR dual responses were fabricated by coating spherical zinc ferrite with poly(*N*-methylaniline) (PMA), thus making a spherical conducting polymer at the same time. Note that, in general, fabrication of sub-micron-sized spherical conducting polymers are very difficult. The E/MR fluids were prepared by dispersing particles with a zinc-ferrite core and an electrically reactive PMA shell in silicone oil. The rheological responses of the final E/MR fluid in which the particles were dispersed to electric and magnetic field stimuli were analyzed [33,34]. The rheological properties of ZnFe_2_O_4_/PMA-based E/MR fluid were investigated using controlled shear and dynamic tests. Shear stress (τ), shear viscosity, yield stress ($\tau_{y}$), and viscoelastic behavior of the E/MR fluid under different *E* and *H* were measured with a rotation rheometer.

## 2. Experimental

### 2.1. Materials

The core was synthesized using FeCl_3_∙6H_2_O (Sigma-Aldrich, Burlington, MA, USA) and ZnCl_2_ (Yakuri Pure Chem., Kyoto, Japan) as the input chemicals of the metal ion source. Ethylene glycol (Daejung Chem., Busan, Korea) and polyethylene glycol (PEG) 400 (DC Chem., Seoul, Korea) were employed as solvents. The surfactant used for fabricating the core was sodium acetate (Sigma-Aldrich, Burlington, MA, USA). *N*-methylaniline (Junsei Chemical, Tokyo, Japan) was adopted for the polymerization of the shell. Ammonium persulfate (Daejung Chemical, Busan, Korea) was purchased as the initiating chemical in the polymerization process. Ethanol (Samchun Pure Chem., Seoul, Korea), HCl (Junsei Chem., Tokyo, Japan), and DI-water were used as solvents. A sodium hydroxide solution (Daejung Chem., Busan, Korea) was introduced for a de-doping step.

### 2.2. Sample Preparation

**Scheme 1:**
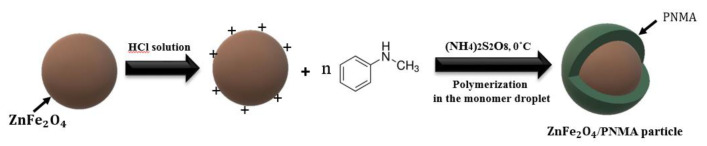
Schematics of the synthetic steps of ZnFe_2_O_4_/PMA microspheres.

Scheme 1 shows the whole step for ZnFe_2_O_4_/PMA core/shell particle synthesis. First, ZnFe_2_O_4_ was fabricated via a solvothermal process. The ZnFe_2_O_4_ surface was protonated using a HCl solution, and *N*-methylaniline was added for chemical oxidative polymerization. An electrostatic interaction occurred between zinc ferrite and aniline.

#### 2.2.1. Fabrication of Poly(*N*-Methylaniline) Coated ZnFe_2_O_4_ Particles

In the experimental process, zinc ferrite was synthesized first and then coated with a conductive polymer. FeCl_3_∙6H_2_O (5.4 g) and ZnCl_2_ (1.36 g) were added to ethylene glycol (200 mL) and stirred with a magnetic bar. After confirming that the solution was mixed homogeneously, sodium acetate (14.4 g) and PEG (4 g) were added and stirred for approximately one hour. The mixture was transferred to an autoclave and baked at 200 °C for 12 h. The synthesized material was cooled, separated using a magnetic bar, and washed several times with ethanol. The obtained products were dried in a vacuum oven for 24 h to obtain dark brown zinc ferrite [35]. The electrical conductivity of ZnFe_2_O_4_ was measured to be 0.12 × 10^−6^ S/cm [36].

PMA was coated on the surface of the initially produced zinc ferrite. A zinc ferrite surface treatment was performed for a uniform polymer coating. Zinc ferrite (2 g) was added to a 0.1 M HCl aqueous solution (800 mL). After 10 h stirring at 5 °C, the products were gathered using a magnetic bar. Anhydrous ethanol (200 mL) and aniline (2 mL) were then input into the reactor. The solution was mechanically stirred at 5 °C for 12 h, and HCl (3.4 mL) was then added. The ammonium persulfate solution (250 mL, 0.04 M) was then input dropwise and stirred for 8 h. The particles were obtained by separating the solution and particles with a magnetic bar. The products were washed several times with ethanol and DI water. The measured electrical conductivity of the obtained particles was 9.33 × 10^−4^ S/cm via a four-probe resistivity tester (MCP-HT450, Mitsubishi Co., Tokyo, Japan). The de-doping process, which is an alkali treatment to lower the electrical conductivity of the PMA part of the synthesized particles by adjusting the pH, was performed using NaOH solution (1 M) at room temperature. The decreased conductivity of the particles could prevent the electrical short circuit during the ER test under the *E* [37]. The products were put in DI water, and sodium hydroxide solution was dropped until the pH reached 8, and the solution was then stirred continuously for 24 h. The final products were then magnetically separated and cleaned with DI water.

#### 2.2.2. Fabrication of E/MR Fluid

To enter the semiconducting range right for ER measurement, controlling the electrical conductivity of the ER materials is required. The de-doping process was performed on the ZnFe_2_O_4_/PMA particles to reduce their electrical conductivity. After this step, their electrical conductivity changed from 9.33 × 10^−4^ S/cm to 4.37 × 10^−7^ S/cm (averaged value of five times test results using each pallet of both ZnFe_2_O_4_ and ZnFe_2_O_4_/PMA). The resulting ZnFe_2_O_4_/PMA particles (5 vol%) were suspended in silicone oil (density: 0.97 g/cm^3^, 100 cSt, KF-96, Shin-Etsu, Tokyo, Japan) for the E/MR fluid. Before the E/MR test, the final suspension was subjected to sonication and vortex mixing to enhance the dispersion.

### 2.3. Characterization

The size and morphological state of the surface of ZnFe_2_O_4_/PMA particles were studied by scanning electron microscope (SEM) (SU-8010, Hitachi, Tokyo, Japan) and transmission electron microscope (TEM) (CM200, Philips, Amsterdam, The Netherlands). The electrical conductivity of the sample pallets was measured using a resistivity meter through the above-mentioned four-point standard prove technique. The chemical structure and compositions of the ZnFe_2_O_4_/PMA were studied by Fourier transform-infrared spectroscopy (FT-IR) (VERTEX 80V, Bruker, Billerica, MA, USA). The crystal information of the ZnFe_2_O_4_/PMA was examined by X-ray diffraction (XRD) (DMAX-2500, Rigaku, Tokyo, Japan). The thermal characteristics of the product were measured via a thermogravimetric analyzer (TGA) (TA Q50, TA Instruments, New Castle, DE, USA) in an N_2_ environment. The chain arrangement of the dispersed particles under an *E* was taken using an optical microscope (OM) (BX-51, Olympus, Tokyo, Japan). The E/MR behaviors of the sample were studied using a Couette rheometer (MCR 302, Anton-Paar, Graz, Austria).

## 3. Results and Discussion

**Figure 1:**
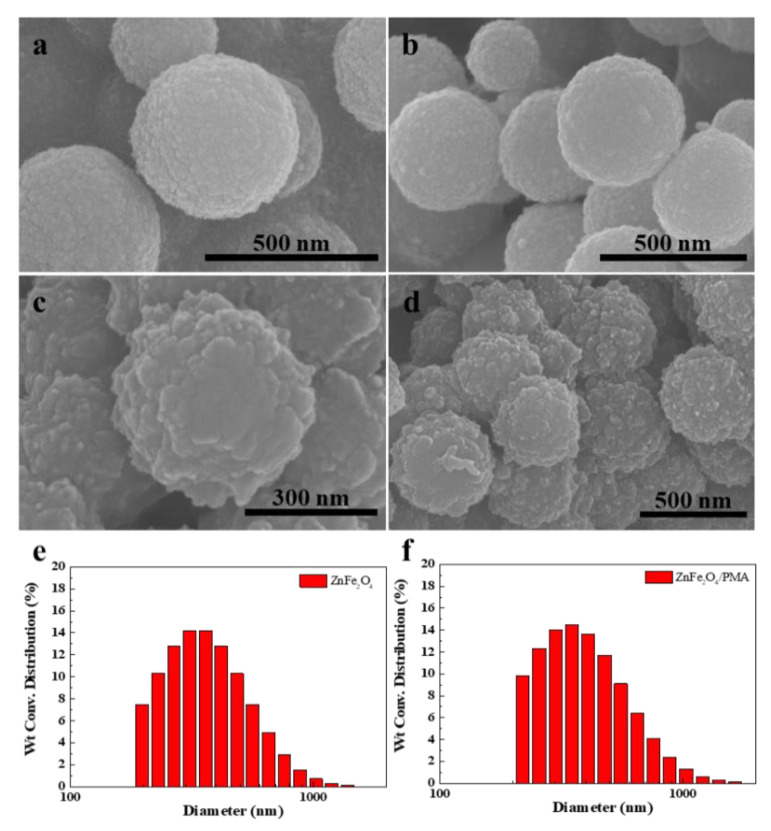
SEM and size distribution of ZnFe_2_O_4_ (**a**,**b**,**e**) and ZnFe_2_O_4_/PMA (**c**,**d**,**f**) core-shell particles.

Figure 1a–d presents the surface morphology of both ZnFe_2_O_4_ and ZnFe_2_O_4_/PMA particles. The ZnFe_2_O_4_ microspheres have a smooth surface. On the other hand, ZnFe_2_O_4_/PMA microspheres have a rougher surface than ZnFe_2_O_4_ microspheres. The ZnFe_2_O_4_ particles were approximately 300–500 nm, and the particles of ZnFe_2_O_4_ covered with PMA were larger than pure ZnFe_2_O_4_. As shown in Figure 1, ZnFe_2_O_4_ and ZnFe_2_O_4_/PMA were synthesized successfully. Figure 1d,e shows the size distribution of ZnFe_2_O_4_ and ZnFe_2_O_4_/PMA particles. The average sizes of ZnFe_2_O_4_ and ZnFe_2_O_4_/PMA were 393.6 nm and 428.2 nm, respectively, and it was confirmed that the size increased after PMA coating.

**Figure 2:**
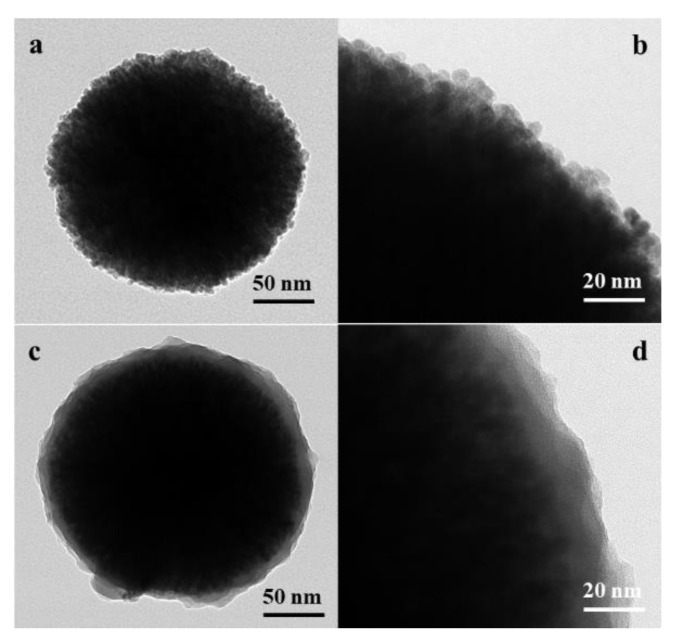
TEM photos of ZnFe_2_O_4_ (**a**,**b**) and ZnFe_2_O_4_/PMA (**c**,**d**) core-shell microspheres.

Figure 2a,b presents the TEM images of ZnFe_2_O_4_. The size of pure ZnFe_2_O_4_ was about 300 nm. Figure 2c,d indicates the shell PMA. The dark spherical state inside the particle is the ZnFe_2_O_4_ portion. The gray area in Figure 2c covers the dark core ZnFe_2_O_4_ with a PMA shell coated on the core. The PMA thickness was approximately 20 nm, as shown in Figure 2d. TEM images of zinc ferrite and ZnFe_2_O_4_/PMA particles clearly show the outer PMA shell surrounding the ZnFe_2_O_4_ core. Compared with the SEM images in Figure 1, the surface features in Figure 2 look different due to the difference in measurement method and resolution.

**Figure 3:**
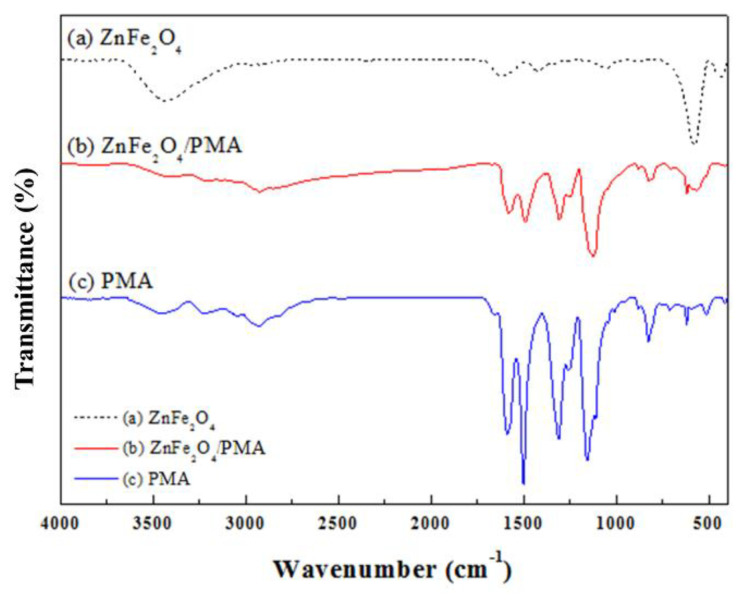
FT–IR spectra of (**a**) ZnFe_2_O_4_, (**b**) ZnFe_2_O_4_/PMA core-shell microspheres, and (**c**) PMA.

The chemical components of the ZnFe_2_O_4_/PMA microsphere were identified by FT-IR spectroscopy. The characteristic absorption bands in Figure 3 show the peaks of (a) ZnFe_2_O_4_, (b) PMA-coated ZnFe_2_O_4_, and (c) PMA, respectively. In Figure 3a, 3432 and 1630 cm^−1^ peaks for O–H bonding, 507 cm^−1^ for the natural vibration frequency of Zn^2+^, and 415 cm^−1^ for the octahedral group of Fe^3+^O^2−^ [38]. The characteristic peaks of the PMA near 2820 and 1302 cm^−1^ were allocated to the NCH_3_ bending vibration and C–N stretching vibration, respectively [39]. The solid red line indicates that the PMA-coated ZnFe_2_O_4_ exhibited absorption peaks for both ZnFe_2_O_4_ and PMA. In the case of ZnFe_2_O_4_/PMA, the ZnFe_2_O_4_-affected peak intensity was weakened by the PMA coating.

**Figure 4:**
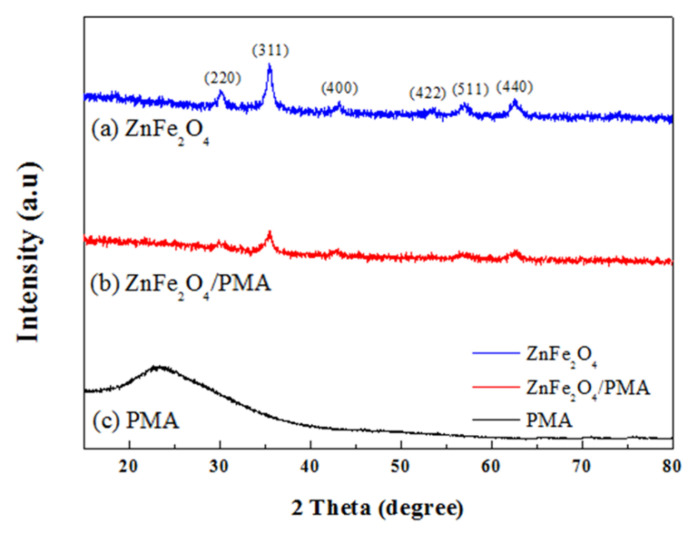
XRD spectra of (**a**) ZnFe_2_O_4_, (**b**) ZnFe_2_O_4_/PMA core-shell microspheres, and (**c**) PMA.

Figure 4 presents XRD patterns of (a) ZnFe_2_O_4_, (b) PMA-coated ZnFe_2_O_4_, and (c) PMA. Figure 4a,b shows the crystal planes in (111), (220), (311), It has (400), (422), (511), (440) and (533) [40]. The crystalline structure of ZnFe_2_O_4_ was maintained, even though the PMA coating process was performed in an aqueous HCl solution. The XRD peak intensity in Figure 4b was lower than in Figure 4a because of the coated amorphous polymeric shell. Overall, the crystal plane of ZnFe_2_O_4_ was well maintained after coating with the conductive polymer PMA.

**Figure 5:**
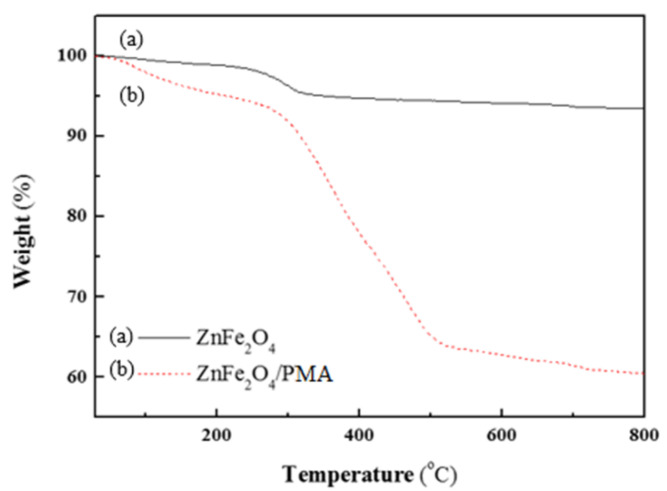
TGA data of (**a**) ZnFe_2_O_4_, and (**b**) ZnFe_2_O_4_/PMA particles.

The thermal behaviors of the particles and the mass percentage of PMA in the ZnFe_2_O_4_/PMA were examined by TGA. TGA confirmed the thermal stability of the ZnFe_2_O_4_ and ZnFe_2_O_4_/PMA particles, as shown in Figure 5. The solid black line in Figure 5a is the curve for ZnFe_2_O_4_, and the red dot line in Figure 5b is the curve of ZnFe_2_O_4_-coated with PMA. In the case of ZnFe_2_O_4_ in Figure 5a, a weight loss of about 3% occurred at about 250 °C, indicating the decomposition of surfactants and residual organic chemicals [41]. As a result of the thermal decomposition of ZnFe_2_O_4_/PMA in Figure 5b, weight loss due to moisture occurred at around 100 °C. After 300 °C, a rapid weight loss of about 30% was observed due to the decomposition of ZnFe_2_O_4_/PMA. The main backbone of PMA surrounding the core started to decompose at a high temperature, and the curve decreased sharply. The final weight contents of ZnFe_2_O_4_ and PMA in the ZnFe_2_O_4_/PMA were 60.5% and 39.5%, respectively.

**Figure 6:**
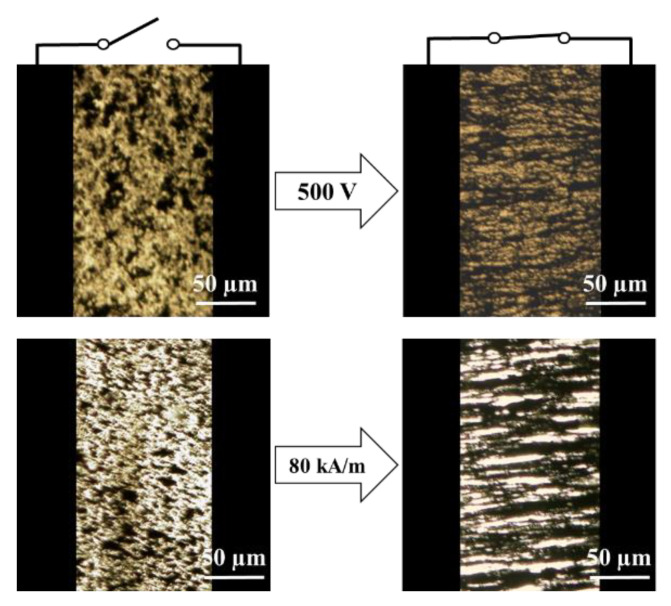
OM images of the ZnFe_2_O_4_/PMA particle-based smart fluid under *E* (**top**) and *H* (**bottom**).

The ER particles were placed irregularly in dispersing medium without a field (Figure 6). By contrast, the particles formed a chain structure when either an *E* or *H* was activated.

**Figure 7:**
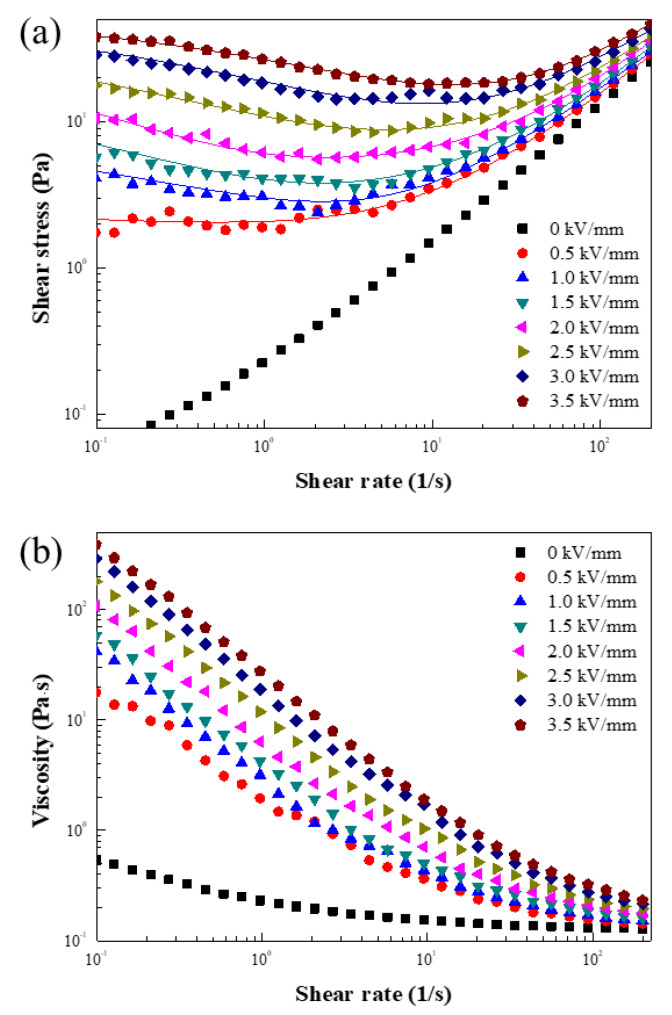
Shear stress (**a**) and shear viscosity (**b**) as a function of $\dot{\gamma }$ for the ER fluid under the *E* from 0 to 3.5 kV/mm (the solid lines in (**a**) are fitted using the CCJ equation).

Before measuring the ER properties, the ER fluid was well mixed using a vortex process. ER characterization was measured while applying an *E* from 0 to 3.5 kV/mm using a rotational rheometer with a shear rate ($\dot{\gamma })$ from 0.1 to 200 1/s. Figure 7a,b shows the τ and shear viscosity (η) curves, respectively. Without an applied *E*, Newtonian-like fluid property was detected with a linear increase in τ as a function of $\dot{\gamma }$. Figure 7a was fitted with the Cho–Choi–Jhon (CCJ) equation [15], using Equation (1).

(1) $$\tau =\frac{\tau_{y}}{1+{\left(t_{1}\dot{\gamma }\right)}^{\alpha }}+\eta_{\infty }\;\left(\;1+\frac{1}{{\left(t_{2}\dot{\gamma }\right)}^{\beta }}\;\right)\dot{\gamma }$$

where $t_{1}$ and $t_{2}$ are time parameters combined with the exponents *α* and *β* controlling the reduction region and increase in the τ, respectively. Table 1 gives the calculated parameters.

While the η as a function of $\dot{\gamma }$ without an input *E* showed a Newtonian-like weak shear-thinning behavior, significant shear-thinning characteristics indicating their solid-like behavior was observed under the applied *E* as given in Figure 7b.

**Figure 8:**
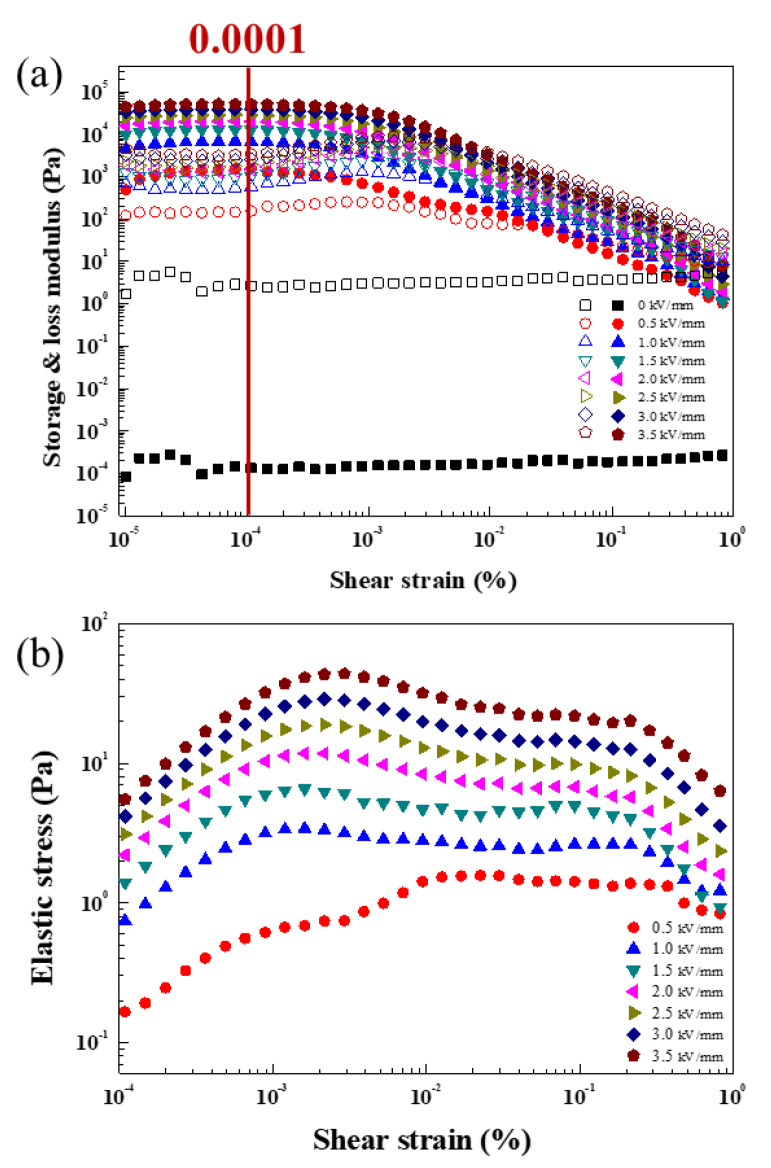
(**a**) Strain amplitude sweep measurement (closed: G′, open: G″) as a function of γ of ZnFe_2_O_4_/PMA based ER fluid and (**b**) τ′ as a function of γ obtained using G′.

The viscoelastic properties of ZnFe_2_O_4_-coated with PMA were carried out using a dynamic oscillation test by controlled shear deformation mode. Figure 8a gives the strain (γ) amplitude sweep measurement result with a set frequency of 1 Hz. The storage modulus (G′) shows the elastic portion, and the loss modulus (G″) implies the viscous portion. In a low strain region, G′ shows a plateau value, called the linear viscoelastic (LVE) range [42], where the chain-like structure is being kept against an input γ. In this area, the elastic contribution is more superior than the viscous contribution, and the structure inside the ZnFe_2_O_4_/PMA-based ER fluid remains unbroken against strain. The strain in the LVE region was set to be 0.0001, as presented in Figure 8a. When the γ becomes larger than its critical value, the formed chain structures began to break, exhibiting a sharp decrease in G′. In Figure 8b, the modulus values obtained from the strain amplitude sweep measurement (see Figure 8a) were scrutinized for the elastic stress (τ′), which can be deduced using the equation τ′ = G′γ. Because the elastic stress is calculated by the strain and G′ values and the elastic yield stress is estimated from its deflection point at the end of the LVE region, obtaining the accurate elastic yield stress point might cause some experimental error. The elastic stress shows elastic deformation of the chain formed between the ZnFe_2_O_4_/PMA particles as the strain increases. The maximum τ′ point is defined as the elastic yield stress, i.e., the maximum limit where elastic deformation is possible [43].

**Figure 9:**
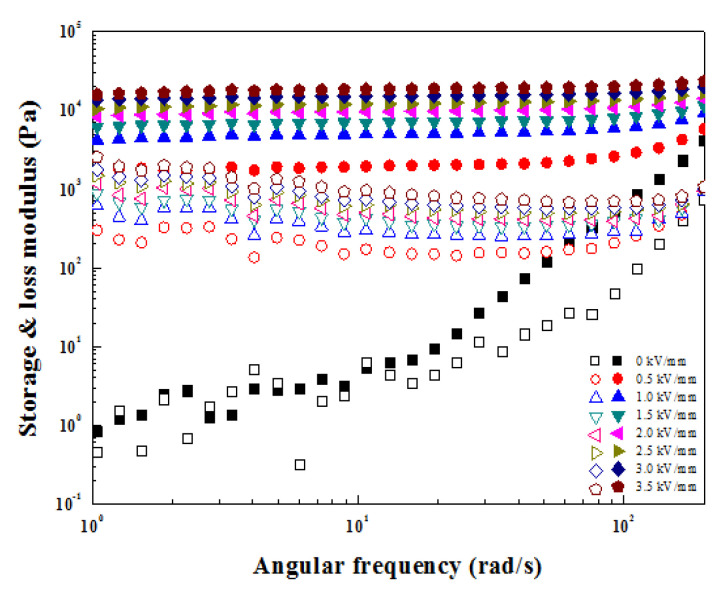
G′ (closed) and G″ (open) in the frequency sweep measurement of ZnFe_2_O_4_/PMA-based ER fluid.

Figure 9 shows the frequency sweep measurements of the ZnFe_2_O_4_/PMA-based ER fluid. The γ value was fixed to 0.0001 from the LVE region set in the strain amplitude test. The frequency ranged from 1 to 200 rad/s, and the applied *E* increased to 3.5 kV/mm. In the absence of an applied *E*, the moduli increased with an increased frequency and exhibited a liquid-like property. In contrast, the moduli increased with increased *E*. Additionally, G′ was higher than G″, suggesting that a strong chain structure was formed and solid-like behavior was observed [44].

**Figure 10:**
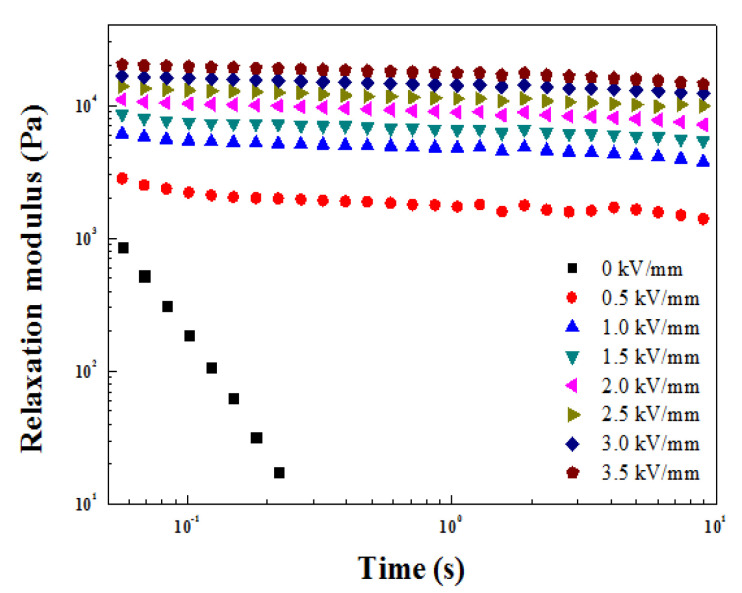
G(t) of ZnFe_2_O_4_/PMA-based ER fluid calculated from G′ and G″ in the frequency sweep measurement.

Through Figure 9, the G′ and G″ according to angular frequency were obtained. The phase change of the ZnFe_2_O_4_/PMA-based ER fluid from solid-like to liquid-like was investigated, as shown in Figure 10. The time-dependent relaxation modulus G(t) was obtained using Equation (2) of the Schwarzl equation [45] and plotted as a function of G′ and G″ deduced from Figure 9. G(t) is a tool to estimate relaxation behavior. Without the electric field, the G(t) decreased rapidly with time and was similar to fluid behavior. With applied *E*, G(t) was relatively constant and exhibited solid-like behavior.

(2) $$G\left(t\right)≅\;G^{\prime }\left(\omega \right)-0.566G^{\prime \prime }\;(\omega /2)+0.203G^{\prime \prime }\left(\omega \right)$$

**Figure 11:**
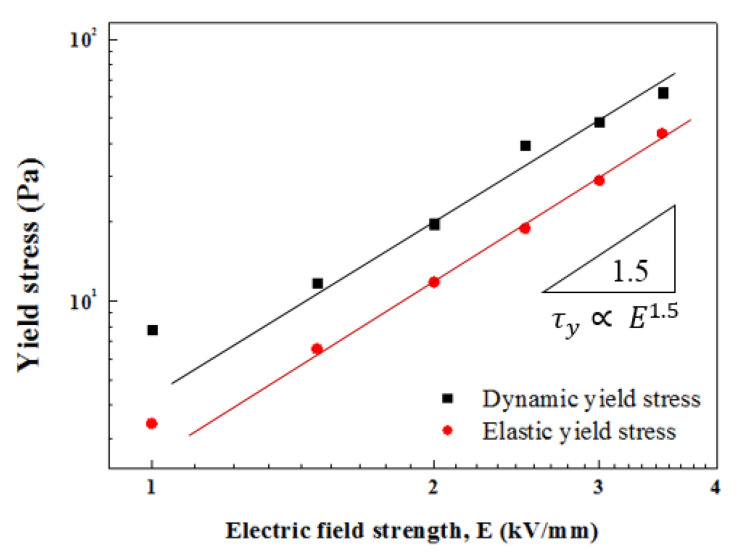
Yield stress as a function of *E* of ZnFe_2_O_4_/PMA-based ER fluid.

The dynamic $\tau_{y}$ obtained from the extremely low $\dot{\gamma }$ limit of the τ is expressed as a function of the input *E* in Figure 11 using a power-law equation (Equation (3)) [46]. Because the slope is approximately 1.5, in the ZnFe_2_O_4_/PMA case, the response implies the conduction mechanism [37].

(3) $$\tau_{y}\propto E^{m}$$

**Figure 12:**
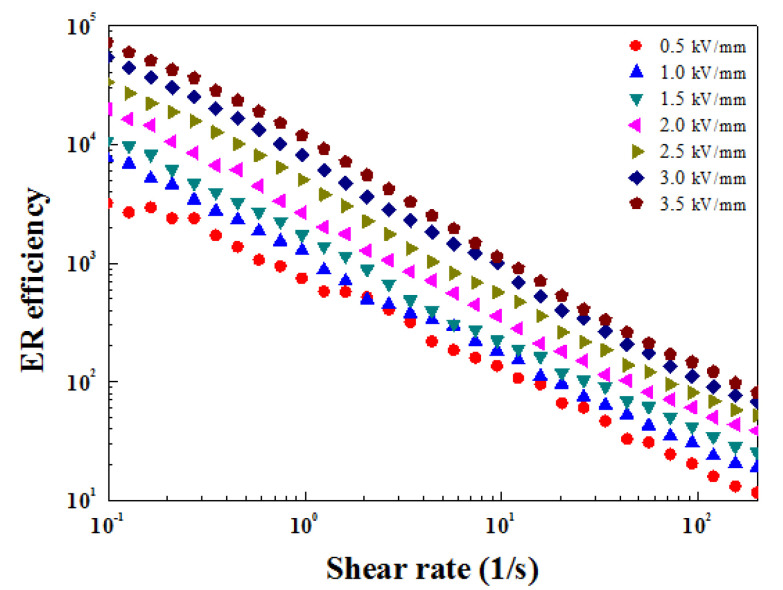
ER efficiency for ZnFe_2_O_4_/PMA-based ER fluid on $\dot{\gamma \;}$ under various *E*.

Figure 12 presents the ER efficiency according to the shear rate for different applied *E*. The ER efficiency was determined from the electrical response value of the ZnFe_2_O_4_/PMA-based ER fluid to the electric field according to Equation (4).

(4) $$e=\frac{\eta_{E}-\eta_{0}}{\eta_{0}}\times 100\;\left(\%\right)$$

when *E* is applied, τ, and shear viscosity are represented by $\tau_{E}$ and $\eta_{E}$. Without an applied *E*, the τ and shear viscosity were $\tau_{0}$ and $\eta_{0}$. In Figure 12, the ER efficiency increased with increased *E*. In addition, the ER efficiency decreased sharply with increased $\dot{\gamma }$. This suggests that gradual collapse of the structure occurs within the ER fluid as the shear rate increases.

**Figure 13:**
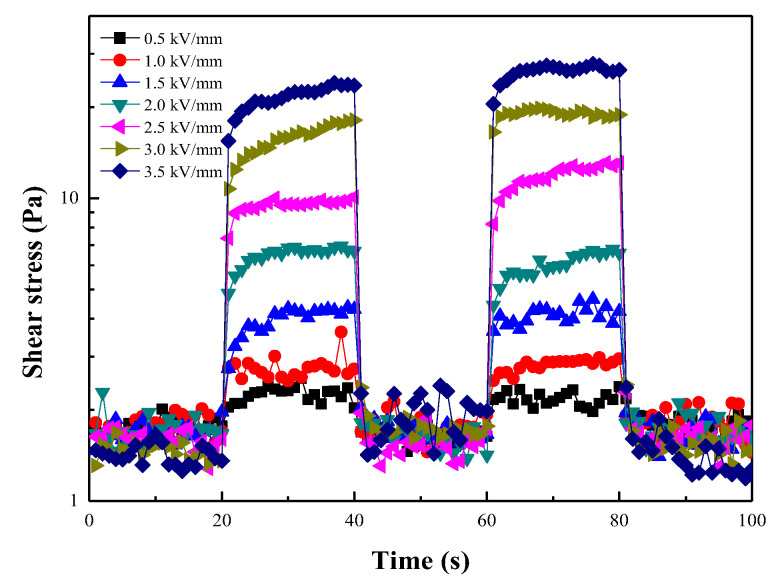
Shear stress of the ER fluid at $\dot{\gamma }=$ 1 s^−1^ under different *E* with a square voltage pulse for 20 s.

Figure 13 presents τ as a function of time to study the stability and sensitivity of the ER fluids under electric fields. $\dot{\gamma }$ was fixed to 1 s^−1^, and an *E* was applied at 20 s intervals. Under the applied *E*, τ increased without hysteresis behavior and decreased immediately to the value of the zero-field state without the *E*. During the on-off switching process of *E*, the liquid-to-solid phase transformation occurred immediately, and the sensitive response of stress to the electric field was confirmed.

**Figure 14:**
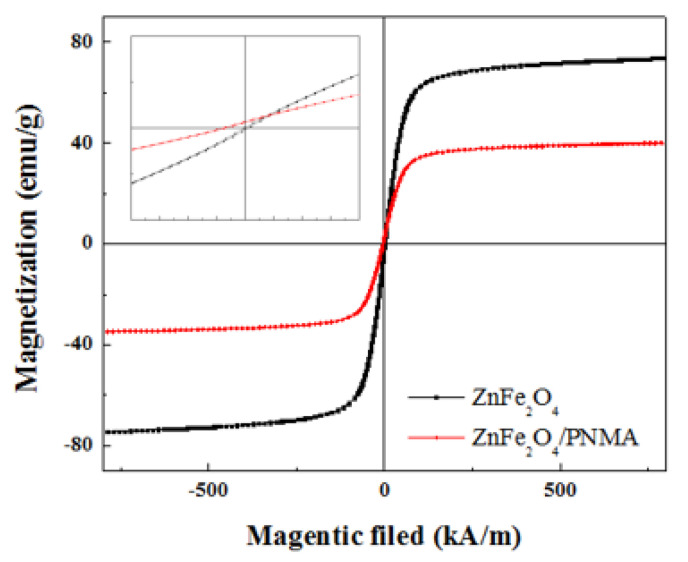
VSM of the ZnFe_2_O_4_/PMA-based 5 vol% MR fluid.

The magnetic hysteresis of the samples coated with zinc ferrite and polymer were measured using a VSM by imposing the *H* from −785 to 785 kA/m at room temperature. As shown in Figure 14, the maximum magnetic saturation of zinc ferrite and the PMA-coated particles according to the magnetic field were 73.67 and 39.93 emu/g, respectively. The coercivity was reduced by coating the non-magnetic PMA.

**Figure 15:**
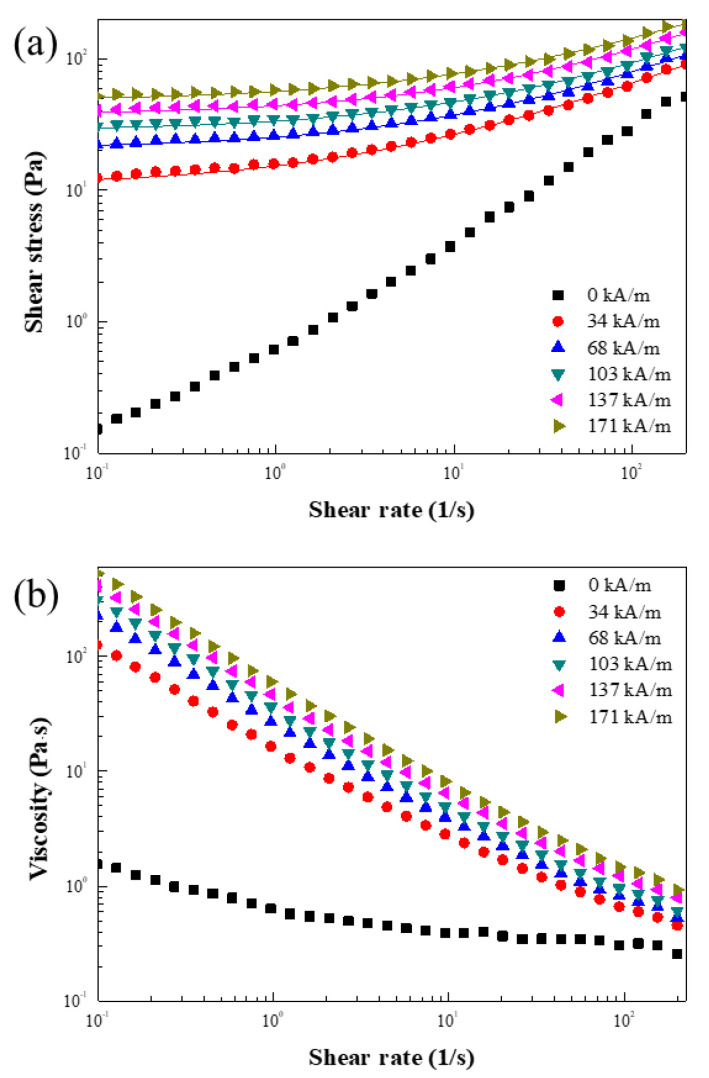
Shear stress (**a**) and shear viscosity (**b**) as a function of $\dot{\gamma }$ for the MR fluid under *H* from 0 to 171 kA/m (The solid lines are fitted using the H-B equation).

Figure 15 shows the MR properties of ZnFe_2_O_4_/PMA particle-based fluids according to the magnetic field strength. The flow curve with $\dot{\gamma }$ from 0.1 to 200 1/s was obtained using the controlled shear rate (CSR) mode. The τ (Figure 15a) and η (Figure 15b) obtained through the measurements were plotted as a function of the $\dot{\gamma }$; both values increased rapidly with increasing external *H*. In addition, as the shear rate increased, interchain breakage due to shear occurred, and the viscosity decreased. Although PMA was coated to partially mask the magnetic properties, it had a stress value similar to that of other MR materials and showed sufficient MR properties [47,48]. The shear stress measurements in Figure 15a fitted the Herschel–Bulkley (H-B) model well. Table 2 lists the results of the parameters. The Herschel–Bulkley modeling equation and the names of each parameter are as follows [49].

(5) $$\tau \left(H\right)=\tau_{y}+K{\left(\dot{\gamma }\right)}^{n}$$

where K and n are the consistency and flow index, respectively. Table 2 lists the parameters obtained from Equation (5).

**Figure 16:**
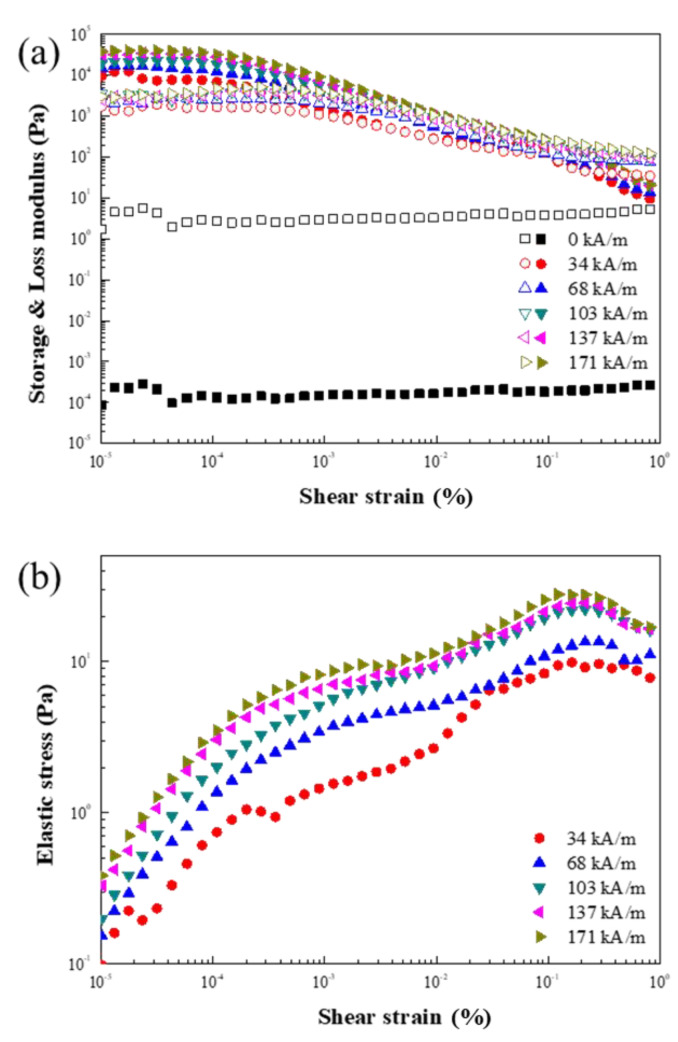
(**a**) Strain amplitude sweep measurement (closed: G′, open: G″) as a function of the strain of ZnFe_2_O_4_/PMA-based MR fluid and (**b**) τ′ as a function of γ obtained using the G′.

Figure 16 gives the dynamic oscillation test results under the magnetic field of ZnFe_2_O_4_/PMA particles based on the MR fluid. Figure 16a,b shows a graph of the strain amplitude sweep measurement obtained from 10^−5^ to 1 of the amplitude strain at a set frequency of 1 Hz and the elastic stress, respectively. The G′ of the strain energy stored and G″ of the strain energy dissipated were measured according to the strain range. The LVE region can be observed, where G′ and G″ are constant. G′ was greatly measured when a magnetic field was applied because the elastic properties dominate the viscous properties because of the chain structure formed inside the MR fluids. On the other hand, G″ had a larger value than G′ at higher amplitude strain. This is because a high strain caused the internal structure to collapse, resulting in viscous-dominated liquid-like behavior.

**Figure 17:**
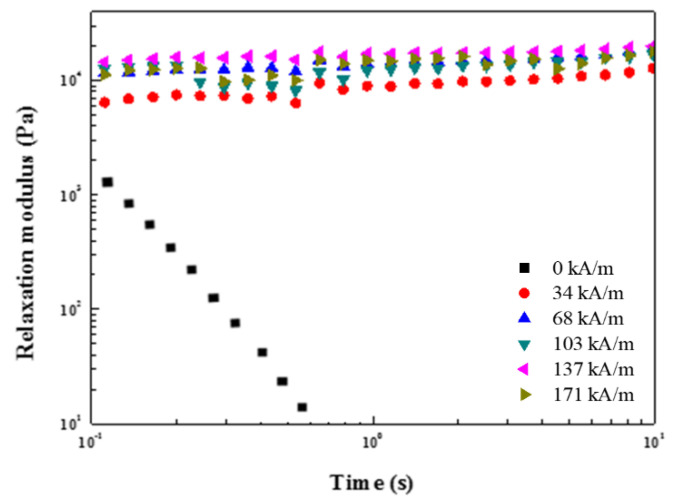
G(t) of the ZnFe_2_O_4_/PMA-based MR fluid analyzed from G′ and G″ in the frequency sweep measurement.

The solid-like characteristics of the MR suspension were analyzed by calculating the shear stress relaxation, G(t) based on the values inferred from the frequency sweep test (Figure 17) using Equation (2), similar to the case of an ER fluid. Without an applied *H*, the G(t) value of the MR fluid decreased with time and exhibited liquid-like properties. The *H* was applied up to 273 kA/m, and the G(t) curve showed no stress relaxation and a stable region with a solid-like property because of the chain-like development formed by strong attraction. Furthermore, G(t) increased as the *H* was strongly applied.

The $\tau_{y}$ was estimated from the flow curves in Figure 15, based on the Herschel–Bulkley equation. The $\tau_{y}$ increased with increasing *H*, and its relationship is expressed as follows:

(6) $$\tau_{y}=H^{\alpha }$$

**Figure 18:**
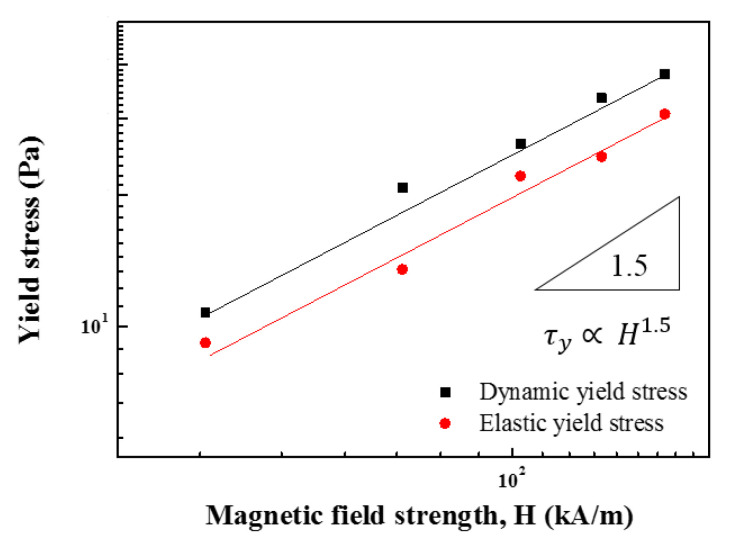
Yield stress as a function of *H* of ZnFe_2_O_4_/PMA-based MR fluid.

Figure 18 shows the correlation between the *H* and $\tau_{y}$ at various *H*. Based on Equation (6), the $\tau_{y}$ increases with increased *H*. The slope was close to 1.5 for both dynamic and elastic yield stresses, i.e., $\tau_{y}=H^{1.5}$.

**Figure 19:**
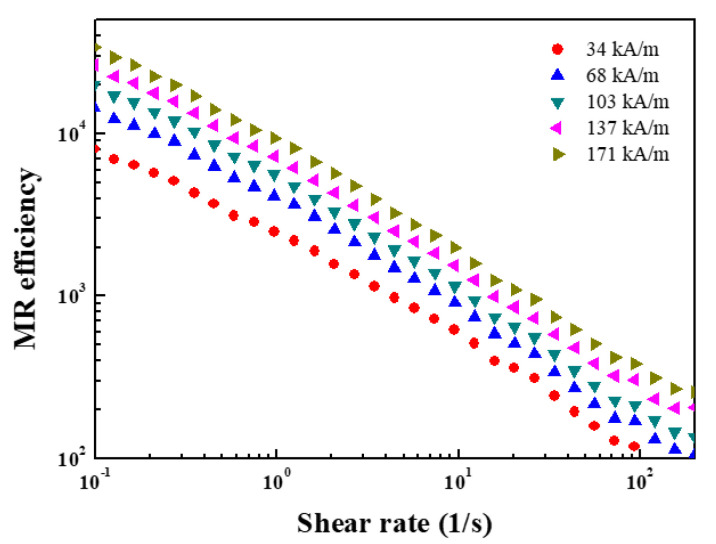
MR efficiency for ZnFe_2_O_4_/PMA-based MR fluid on $\dot{\gamma }$ under different *H*.

The magnetic response of the ZnFe_2_O_4_/PMA particle-based MR fluid was examined by estimating the MR efficiency using Equation (7).

(7) $$e=\left(\eta_{H}-\eta_{0}\right)/\eta_{0}\times 100\;(\%)$$

where $\eta_{H}$ are the shear viscosity under an input *H*, respectively. $\eta_{0}$ is the shear viscosity in the absence of an applied *H*. In Figure 19, the MR efficiency decreased sharply with increasing $\dot{\gamma }$, suggesting that gradual collapse of the structure within the MR fluid occurs as the shear rate increases.

Finally, it can be further noted that the ER performance of the ZnFe_2_O_4_/PMA-based ER fluid in this work is comparable to that of previously reported PMA-based ER fluids (our yield stress with 5 vol% is 50 Pa, while 10 vol% from Ref. [50] is 103 Pa and 5 vol% from Ref. [51] is about 50 Pa) even though their direct comparison is not easy because particle concentrations and particle size and shape of these ER fluids are not exactly same. Nonetheless, the merit of this work is that by producing the core-shell structured ZnFe_2_O_4_/PMA particles, the amount of the PMA shell portion synthesized is extremely small compared to the whole pristine PMA particles in the previously reported ER fluids [50,51]. The availability of fabricating almost spherical and monodispersed PMA particles is another benefit in the case of the core-shell structured ZnFe_2_O_4_/PMA particles compared to the irregular shaped and polydispersed PMA particles in the previous works [50,51]. Furthermore, their dual response along with the MR characteristics due to the ZnFe_2_O_4_ core part is another outcome compared to the previous PMA-based ER fluids even though the MR property of this work can not be compared to the carbonyl iron-based MR fluid because of the big difference in their saturation magnetization values. It can also be noted that that the simultaneous applications of both electric and magnetic fields can enhance their yield stress [52] even though the yield stress we have is rather small due to its low particle concentration. While we put our concentration on the dual response itself with a rather low particle concentration, its yield stress will be increased a lot for a higher concentration. Even with a rather low yield stress, there are areas for its potential applications such as optical [53] and tactile [10] display.

## 4. Conclusions

Magnetite/semiconducting polymer composite particles were synthesized with the merits of better controlling their shape, monodispersed size, and dual electric and magnetic functionality. A core-shell structure with a rough surface compared to the pristine zinc ferrite was characterized by SEM, and relatively uniform coating thickness was confirmed by TEM. The particle size was 300–500 nm, and the thickness of the coated PMA was 20–40 nm. FT-IR spectroscopy confirmed the chemical compositions of the ZnFe_2_O_4_/PMA and XRD verified the crystal face of the core ZnFe_2_O_4_. The de-doping process finally confirmed an electric conductivity of 4.37 × $10^{-7}$ S/cm. The core ZnFe_2_O_4_ density was about 4.8 g/cm^3^ and the ZnFe_2_O_4_/PMA particle density was about 2.1 g/cm^3^ with their potential benefit of the sedimentation problem. The rheological properties of the E/MR fluid-based on zinc ferrite coated with PMA were analyzed using a rotating rheometer. The CCJ equation is found to be suitable under various *E*. In the MR test, the magnetic field was applied from 0 to 171 kA/m and was well adapted to the Herschel–Bulkley model. Overall, ZnFe_2_O_4_/PMA particles exhibit stable and tunable dual rheological responses in both magnetic and electric fields, showing that they are potential E/MR materials for E/MR fluid-based applications.

## Figures and Tables

**Table 1:** Parameters of the CCJ equation fitting deduced from ZnFe_2_O_4_/PMA−based ER fluid.

Model	Parameters	Electric Field Strength (kV/mm)						
		0.5	1.0	1.5	2.0	2.5	3.0	3.5
**Cho-Choi-Jhon**	$\tau_{0}$	3.08	7.80	11.69	19.74	39.52	48.43	52.57
	$\eta_{\infty }$	0.10	0.09	0.14	0.14	0.13	0.06	0.07
	α	0.19	0.46	0.70	0.82	0.46	0.47	0.46
	β	0.30	0.43	0.83	0.90	0.66	0.42	0.36
	$t_{1}$	0.16	5.54	11.56	15.38	15.21	3.44	1.13
	$t_{2}$	0.15	0.03	0.04	0.02	0.01	0.01	0.07

**Table 2:** Parameter of H-B equation fitting deduced from ZnFe_2_O_4_/PMA-based MR fluid.

Model	Parameters	Magnetic Field Strength (kA/m)				
		34	68	102	137	171
**Herschel–Bulkley**	$\tau_{y}$	10.73	20.80	28.31	37.43	47.75
	K	4.65	4.69	5.41	6.59	8.89
	n	0.54	0.55	0.54	0.55	0.52

## Data Availability

Not applicable.
